# Complexity of leaf surface texture affects microbial colonization in temperate forest tree species

**DOI:** 10.1371/journal.pone.0349938

**Published:** 2026-05-29

**Authors:** Waltraud X. Schulze, Ernst-Detlef Schulze, Susanne Reiße, Roman Rischke, Olivier Bouriaud, Burkhard Büdel, Tatsiana Straub, Evelina Pillai, Benjawan Tanunchai, Witoon Purahong, Stefan Simm, Matthias Noll

**Affiliations:** 1 Department of Plant Systems Biology, University of Hohenheim, Stuttgart, Germany; 2 Center of Excellence GreenRobust, University of Hohenheim, Stuttgart, Germany; 3 Max-Planck-Institute for Biogeochemistry, Jena, Germany; 4 Core Facility Hohenheim, University of Hohenheim, Stuttgart, Germany; 5 Department of Applied Natural Sciences and Health, Coburg University of Applied Sciences and Arts, Coburg, Germany; 6 Stefan cel Mare University of Suceava, Suceava, Romania; 7 RPTU Kaiserslautern, Department of Biology, Kaiserslautern, Germany; 8 Department of Soil Science, Helmholtz Centre for Environmental Research, Halle, Germany; Universitat Jaume 1, SPAIN

## Abstract

Throughout their life cycle, tree leaves are subject to colonization and degradation by microorganisms, including fungi, bacteria, and algae. These relationships co-evolved with chemical properties, leaf shape, and surface structures. Here we developed (i) a novel quantitative trait describing leaf surface texture complexity based on variables extracted from scanning electron microscopic images, resulting in a quantitative score of surface texture complexity on a tree species level. This complexity score was then used (ii) to test functional hypotheses, quantifying the contribution of leaf surface texture complexity in context of growth habitat preferences and colonization patterns by fungi and bacteria. We show that (iii) leaf surface texture complexity correlated with anatomical features such as stomatal density and leaf orientation as well as with Ellenberg temperature habitat indicator. Increasing leaf surface texture complexity was negatively correlated with leaf-associated fungal and bacterial specialists. Moreover, leaves with higher leaf surface texture complexity values showed reduced richness of colonization with plant pathogens (broad-leaved species) or lichenization (conifers), suggesting protection effects. Our results highlight leaf surface texture complexity as a previously underappreciated trait that may be a key to understanding microbial diversity between tree species and interaction patterns with leaf-associated microbes. This opens promising avenues for future research on plant-microbe co-evolution, trait-based ecosystem modeling, and the potential use of surface traits in forest management and disease resistance strategies.

## Introduction

The textural complexity of leaves in vascular plants has fascinated botanists since the early use of Scanning Electron Microscopy (SEM) [1, 2]. Plant scientists have used these advances in microscopy to study evolution, function and patterning of the stomata, which regulate gas exchange between the leaf interior and the atmosphere [3]. At the same time, leaves and needles offer a variety of habitats for microorganisms, including bacteria, fungi [4], and algae [5], which colonize the leaves from bud break until leaf senescence [6]. Boosted by advances in sequencing technologies, investigations of the “jungle” of microbial and algal communities that inhabit leaf surfaces [5, 7] have become an important research focus of plant and microbial science in recent years.

Stomatal and cuticular features are likely co-evolutionary adaptations shaped by respective growth habitats. This process is believed to have begun for gymnosperms approx. 298 million years ago, and for angiosperms approx. 200 million years ago [3]. The colonization of leaf surfaces by microbial communities is also presumed to have evolved in a host– and environment-specific manner [8]. Consequently, leaf surface structures may have become key determinants in shaping these organismic interactions. Generally, the ecological roles and functional traits of microorganisms on and within fresh leaves range from pathogenic to mutualistic [9], and fungi are able to shift their ecological roles depending on habitat conditions [6]. Fungal epi– and endophytes were suggested to be the primary saprotrophs following leaf senescence and abscission [4, 9].

Interestingly, a variety of bacteria, oomycetes, and fungi exploit stomatal openings as major invasion routes [10–12] primarily due to the availability of moisture and possibly inorganic ions. To prevent microbe invasion, plants recognize the so-called microbe-associated molecular patterns (MAMPs) which are highly conserved within a class of microbes, such as flagellin for bacteria and chitin oligosaccharides for fungi. The detection of these molecules then triggers stomatal immunity and defense responses, including stomatal closure or inhibition of stomatal opening [13–15]. In turn, some plant pathogens have devised various strategies to manipulate stomata behaviour, facilitating easier invasion [12, 16].

While the composition of microbial communities on leaf surfaces was studied intensively [17], the role of structural, chemical, and elemental leaf surface features has received less attention, although they provide important factors driving colonization [18]. Moreover, methods of implementing leaf surface structures on microscopic level as quantitative variables were developed only recently with advanced imaging and image analysis technology. For example, microscopic leaf surface analysis has been applied to the taxonomic identification of plants [19], focusing primarily on four image features such as shape, texture, vein patterns, and color. Another classification approach utilized epidermal texture patterning by extracting texture features from microscopic images. These features enabled quantitative comparisons based on structural properties, which have led to a significant increase in taxonomic classification of plant species [20]. The classifications based on preprocessed leaf surface images offer the opportunity for higher throughput compared to traditional morphometry studies using transversal leaf cuts [21, 22]. The advantage of such leaf texture classification lies in its ability to exclude noise and artifacts during segmentation, which otherwise could affect the classification outcome. Application of the noise exclusion can result in over 90% correct species classification [20].

On a macroscopic scale of forest landscapes, structural features, such as tree diameter, tree height, dead wood volume, and diversity of bark types, were shown to have high explanatory power for the species richness, especially for plants and some insect groups [23]. On the scale of individual organs, the biochemical properties of leaves, such as reflectance and transmittance spectra, were recently used to classify leaf phenotypes for cultivar classification and selection during breeding [24]. Interestingly, the leaf spectral emissions are also affected by leaf surface properties [25]. However, the lack of sufficiently large-scale quantitative data on leaf epidermal surface characteristics has so far slowed the progress in this research direction [26]. However, leaf surface structural features have not yet been widely exploited under consideration of how these structural features could affect the biological function of the leaves and/or interactions between host plant and organisms living in the phyllosphere (e.g., microbes).

**Fig 1:**
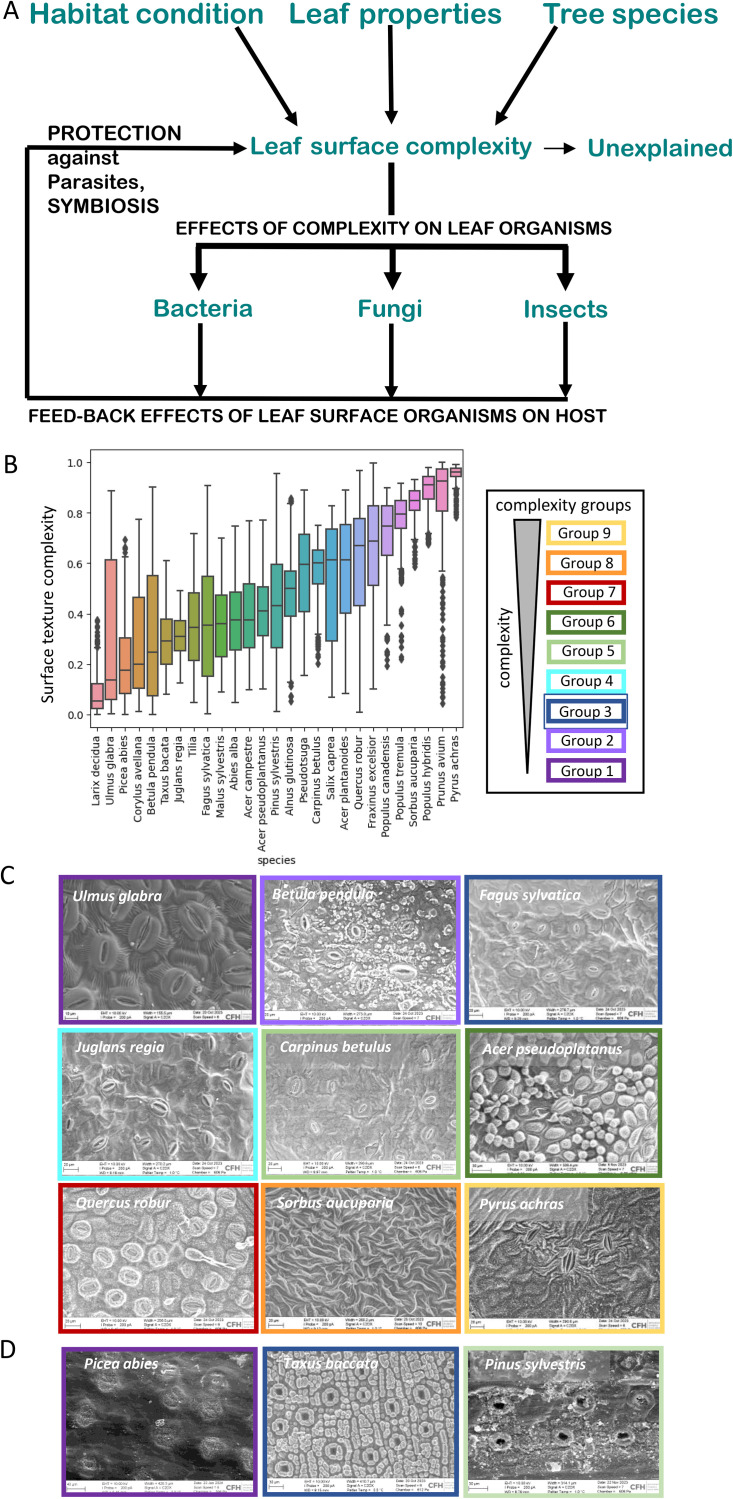
Texture complexity of abaxial leaf side across 27 tree species. (A) Experimental design based on hypotheses tested regarding tree and habitat characteristics affecting surface texture complexity. Leaf texture complexity in turn may affect colonization by various organisms, which may then feed back on tree function. (B) Ranking of texture complexity scores across sampled species. Complexity groups were defined by ANOVA hierarchical clustering. (C) Images of representative broad-leaved species within the nine complexity groups. (D) Images of representative coniferous species from different complexity groups.

Here, we used machine learning methods to quantify leaf surface features from microscopic images of abaxial leaf epidermal surface and (i) developed a ranking of texture complexity based on game-theoretic approaches [27, 28]. The development of a quantitative complexity measure is expected to add explainability to species classification and enable (ii) testing of functional hypotheses regarding the role of leaf epidermal surface texture complexity for leaf functions in dominant tree species of a European temperate forest (Fig 1A). In the following, we firstly hypothesize that leaf surface texture complexity is correlated to evolutionary drivers, such as leaf orientation and habitat preference. Secondly, we hypothesize that leaf epidermal surface texture complexity may determine the diversity of microbial community composition and other organismic interactions (Fig 1A). This is an exploratory study in which we used the newly developed surface texture complexity score to investigate the effects of leaf surface structural complexity on interactions with leaf-associated microorganisms on the scale of different tree species.

## Materials and methods

### Study site and sampling

The mature leaves and needles were collected with permission of the owner (Detlef Schulze, a co-author of this study) from a privately managed mixed temperate forest in the Hainich-Dün region of Thuringia, Germany (51°120N 10°180E). The forest site is located at about 500 m elevation with 600–800 mm annual precipitation and 6 to 7.5 °C mean annual temperature (based on German climate data, DWD www.dwd.de). The bedrock is limestone with 30 cm – 1 m of cambisol soil mainly from weathering (see also [4]). Leaves and needles (S1 Table) were sampled from the ground up to 6 m at the sun-exposed southern edge of a tree crown using a telescopic leaf cutter. Host trees were canopy trees of >25 cm breast height diameter of variable age (60–100 years) due to selective harvest. Canopy height is about 30 m [29]. Most of the sampled tree species were collected in a single stand of about 5 ha. Several leaves of up to five trees per species were collected; sampling was conducted in batches throughout the season. For conifers, fresh needles from the current growing season were sampled. Thus, we examined a forest community where trees interact at the canopy level to resolve leaf surface structures independent of variation in site conditions. For microbiome analysis, a minimum of 200 g leaves and needles per tree individual were collected (each with five true replicates (tree)) in October 2019, with new gloves in sterilized plastic bags. The samples were transported on ice within 3 h to the laboratory and frozen at −80°C before further processing. For microscopy, leaves were collected from the same site in October 2024. Leaves were analyzed fresh, but also after freezing them as part of storage at −80°C. Freezing did not impact the SEM microscopic analysis. Nutrient analysis and DNA extraction were always performed on frozen material. Data from all leaf samples were analyzed together.

### Scanning Environmental Microscopy (SEM)

Surface structures of the leaves were examined using a scanning electron microscope (EVO15, Carl Zeiss Microscopy GmbH). Fresh leaf material from 5 to 20 different leaves was analyzed without fixation or coating with conductive materials, and under conditions of significantly reduced chamber vacuum (610 Pa). Images were collected at two different magnification scales (1900x / 230x) (S1 Fig). Additionally, the instrument was equipped with an Energy Dispersive X-ray Spectroscopy (EDS) detector (UltimMax, Oxford Instruments), enabling elemental analysis alongside and overlaying the imaging. The distribution of C, N, and O in the surface structure was determined for selected species covering a range of leaf surface texture complexity classes, which we derived from our developed protocol (see below). Element distribution was then quantified using AZtec software Version 6.0 (Oxford Instruments).

### Confocal microscopy

Cell walls of hyphae were stained by Calcofluor White and analyzed by confocal microscopy (Zeiss LSM 900). Calcofluor White stains beta-1,3 and beta-1,4 polysaccharides, which interact with chitin or peptidoglycan structures [30, 31]. Each needle or leaf section was incubated in the staining solution (Calcofluor White diluted 1:1 with 10% KOH, [vol/vol]) for 20 min in the dark, followed by rinsing with water. A laser with a wavelength of 405 nm was used to excite the Calcofluor White-colored structures and the fluorescence was detected in the emission range from 405 to 460 nm. The autofluorescence of the chlorophyll was generated with a laser with a wavelength of 640 nm and detected in the range from 640 nm to 700 nm. Images were processed using the LSM Plus deconvolution algorithm and analyzed and exported with the ZEN 3.6 blue edition software (Carl Zeiss Microscopy GmbH).

### Quantification and classification of leaf surface texture complexity

The development of this quantitative assessment of leaf surface texture complexity features is considered a central result of this work. Therefore, further details of this newly developed approach are outlined in the Results and Discussion sections, while technical details are mentioned here in the Methods section.

SEM images were used as databases to quantify the surface texture complexity of leaf abaxial surfaces. For analysis of leaf structural complexity, images from leaf areas without fungal colonization were used. All images from all species and all individual sampled trees were analyzed together to achieve species-level texture signatures. The primary stages of our analysis include sub-image sampling, feature computation, classification of species, and complexity ranking. For sub-image sampling, a fine-tuned model, Detectron2 [32], was applied for precise segmentation of stomata within the SEM images. This targeted approach helped in isolating key features, such as structural variations of wax formation, relevant to the texture complexity analysis. We then randomly sampled 120 sub-images around the identified stomata within a larger image, each of size ca. 80 x 80 µm^2^. Sample images were resized to 512 x 512 pixels to ensure uniformity in feature extraction and analysis. Balancing the number of samples per species results in a dataset of 12960 images. Texture features were computed for each image using the Gray-Level Co-occurrence Matrix (GLCM), spectral features using Fourier transform techniques, combined with Principal Component Analysis (PCA) to distill the images down to 25 significant spectral features, gradient-based attributes, and the compressed size of the image files (S2A Fig). The latter provides insights into the inherent texture complexity of the image data due to the lossless coding. For classification of leaf surfaces from tree species based on the computed features, we utilized the k-nearest neighbors (kNN) algorithm with k = 3. This method has been chosen based on preliminary tests indicating its effectiveness for our dataset and the performance matched previously reported results [20].

Ranking of leaf surface texture complexity features from different species was achieved by a game-based approach (S2B Fig), which was used for its versatility, scalability and the possibility of simple interpretation and an easily performed all-against-all comparison. This game utilized a set of interpretable and uncorrelated features (image compression size, ASM, and gradient standard deviation), each contributing uniquely to the perceived complexity of a leaf. In the game, individual leaf samples were pitted against each other in a pairwise comparison format. During each match-up, the sample exhibiting greater texture complexity, as determined by our set of defined features, was awarded a point, while the other sample lost a point. This dynamic scoring method ensured that the contribution of each feature to overall texture complexity was considered, allowing for a nuanced ranking of leaf/needle surface texture complexity on species-level. We applied the Bradley-Terry model [27] to compute a single score for each species. Species were grouped into distinct classes using hierarchical clustering based on a pairwise comparison with the Kruskal-Wallis test. Overall, the here developed surface texture complexity score resulted in a classification accuracy of 96%, aligning with previously reported results [20]. We chose our training-validation process to be aware of intra-class variation [33] by balancing our dataset through the use of multiple images of different areas per individual leaf, in addition to different leaves.

### Leaf parameters

Stomatal density was calculated from the SEM images. Leaf angle was measured in the field using an inclinometer. Ellenberg indicator values (using numeric scale, typically between 1 and 9) were used as a standardized ecological ranking system to describe the preferred environmental habitat conditions — such as light, moisture, and soil nutrients—of vascular plant species based on vegetation survey [34]. The indicator value for temperature for instance describes the habitat preference (abundance) of species in terms of temperature climate (boreal versus mediterranean) in the present vegetation. The Ellenberg habitat indicator values of tree species under consideration in this study were obtained from a database [35]. Nutrient contents of leaves from the sampled trees were already published previously [36]. Other tree parameters, such as rooting depth, rooting type and mycorrhization listed in S2 Table were derived from literature [37, 38].

### DNA extraction and Illumina sequencing

Detailed methods on DNA extraction and Illumina based MiSeq Sequencing of mature leaves and needles were previously described elsewhere [36, 39]. Up to 10 leaves or needles per tree were sampled from five individual trees. Initially, leaves were carefully rinsed with sterile Tween solution (0.1% vol/vol) and deionized water to remove particles and loosely attached organisms, as explained earlier [39]. As no mechanical shear forces were applied to detach epipihytes [40, 41], microorganisms attached to the surface likely remained unaffected. Tissue grinding recovers combined epiphytic and endophytic communities with higher yield but reduced compartment specificity [42]. Therefore, we refer to the community as leaf-associated microbiome. Samples were ground by using liquid nitrogen and a pestle, homogenized, and stored at −20 °C. For DNA extraction, approximately 120 mg of homogenized leaves or needles were processed using the DNeasy PowerSoil Kit (Qiagen, Hilden, Germany) following the manufacturer’s instructions. Resulting environmental leaves or needles DNA extracts were used for fungal internal transcribed spacer region (ITS)-based and 16S rRNA gene-based amplicon sequencing using the Illumina MiSeq platform as described previously [43]. In this study, fungal and bacterial amplicon sequencing data were generated for only 12 host tree species, whereas surface texture complexity was assessed for 27 species. The subset of 12 tree species covered the full range of surface textures complexity scores observed across all 27 tree species (S3 Fig).

### Bioinformatics and classification of amplicon sequence variants (ASVs)

ITS- and 16S rRNA gene-based sequence dataset obtained on a subset of the twelve tree species (8 broad-leaf, 4 conifers; [39]; supplementary S3 Table). The same trees were sampled for sequencing (in previous work) and for complexity analysis (this study). The ITS- and 16S rRNA gene-based sequences were processed to amplicon sequence variants (ASVs) as explained earlier [4]. Ultimately, 2451 and 1946 rarefied fungal and bacterial ASVs were obtained, respectively. The minimum sequencing depth of 21,967 (fungi) and 15,213 (bacteria) sequences per sample was used. The fungal ecological function of each ASV was assessed using FungalTraits [44]. The taxonomic annotation and quantitative abundance analysis could be biased towards fungal and bacterial species of higher general interest, like pathogens. The classification of generalists and specialists of a taxonomic table was calculated based on the deviation of niche width indices (Shannon, Levins, or occurrence) from null values computed with permutation algorithms for community matrices using the spec.gen function in the EcolUtils package in R (https://github.com/GuillemSalazer/EcolUtils [45]). We classified the leaf colonizing fungi and bacteria as specialists when they were found on only one target tree species, as generalists when they were found common on all tree species, or as opportunists growing selectively on conifers or broad-leaved trees. In total, 2% of the ASVs in the data set [6] can be considered as referring to endophytic organisms.

### Microbial diversity calculation

The Shannon diversity index was computed for fungal and bacterial data set which represents the presence/absence of all species observed on the leaves of the tree species studied [6, 36], all leaves of a given tree species being pooled. The Simpson index was also computed based on the same data set. Because it rendered very similar results we kept one single index of diversity in our analyses, namely the Shannon index, calculated using the diversity function of the vegan 2.6–8 package in R.

### Multivariate analyses and regression analysis

**Fig 2:**
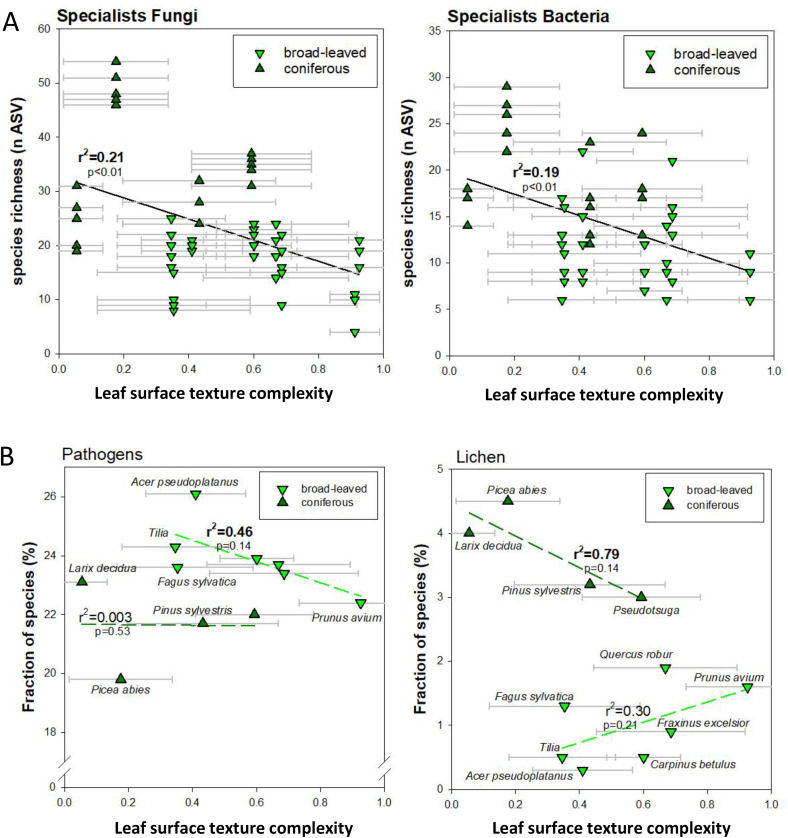
Relationship of fungal and bacterial richness with leaf surface texture complexity. **(A)** Correlation of richness in fungal and bacterial specialists with leaf surface texture complexity. **(B)** Correlation of the proportion of fungal pathogens or lichen-based fungi with leaf surface texture complexity. Data points represent averages with standard deviation. Species richness was assessed by amplified sequence variants (ASVs). P-values and r^2^-values were derived from a multivariate regression analysis using Excel Data Analysis Tool Pack.

In the multivariate regression analysis, we used specific parameters like fraction of microbial species (in percentage), species richness (counted as ASVs), proportion of fungal ecotypes, leaf parameters (leaf angle, stomatal density), or Ellenberg indicator values in relation to the calculated and described leaf surface texture complexity. The multivariate regression is using the least squares method to model the relationship between the dependent variable (species richness or fraction of species, fungal ecotypes, leaf parameters) in relation to the predictor (leaf surface texture complexity) and was computed using Excel Data Analysis Tool Pack Add In (e.g., in Figs 2, S5, S6). The analysis was separated for broad-leaved and coniferous tree species. Single linear regression analysis (e.g., Fig 4) was performed in SigmaPlot v.11.

**Fig 3:**
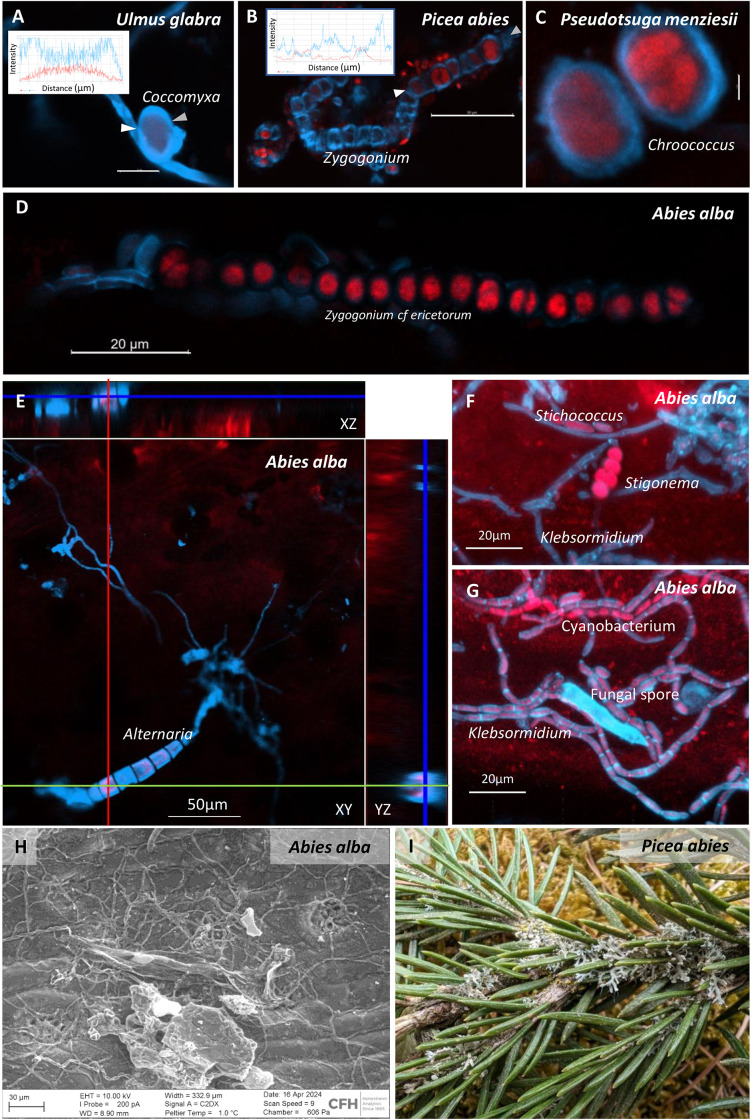
Association of fungi with algae and cyanobacteria. (**A**) Phagocytosis of the algae *Coccomyxa*. Scale bar 5µm. (**B**) Threads of the green algae *Zygogonium*. Scale bar 50µm. (**C**) Unicellular cyanobacteria. *Chroococcus* Scale bar 2µm. (**D**) Threads of green algae *Zygogonium*. Scale bar 20µm. (**E**) Fungal hyphae and spores of *Alternaria* form a layer above the epidermal cells as indicated by XYZ ortho projections. Scale bar 50µm. (**F**) Cyanobacterium *Stigonema* as well as green algae *Stichococcus* and *Klebsormidium*. Scale bar 20 µm.(**G**) Network of algae, cyanobacteria and fungi. Scale bar 20 µm. (**H**) Hyphae form a network between epistomatal wax plates. Scale bar 100 µm. (**I**) *Anaptychia ciliaris* (dominant), *Candelariella xanthostigma* (yellow spots, rare species), *Melanohalea exasperatula* (brown-green layer), and *Apatococcus lobatus* (green algae) growing on 3-year-old needles of *Picea abies* (identification by Burkhard Büdel). In A to G, red shows chlorophyll autofluorescence, cyan shows chitin or peptidoglycan in cell walls stained by Calcofluor White (LSM900, Carl Zeiss Microscopy GmbH). Inserts show fluorescence intensity profiles of calcofluor white (cyan) and chlorophyll (red) along a line between white and gray arrows.

**Fig 4:**
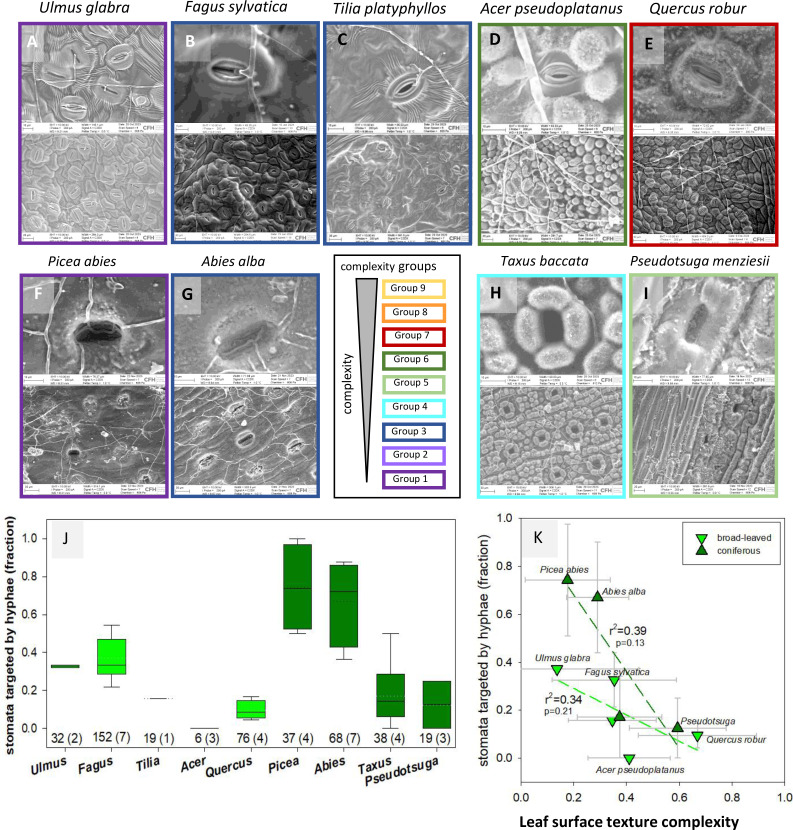
Fungal hyphae growth is directed towards stomata. Broad-leaved species: **(A)** *Fagus sylvatica*, **(B)** *Ulmus glabra*, **(C)** *Tilia platyphyllos* from different texture complexity groups. Protective structures around stomata prevent fungal penetration in **(D)** *Acer pseudoplatanus*, **(E)** *Quercus robur.* Coniferous species: **(F)** *Picea abies*, **(G)** *Abies alba* stomata are heavily targeted by hyphae, while in **(H)** *Taxus baccata* and **(I)** *Pseudotsuga menizesii* protective structures were observed. Images were acquired on a scanning electron microscope (EVO15, Carl Zeiss Microscopy GmbH), border colors indicate respective ANOVA complexity groups. Representative examples are given at closeup magnification of single stomata and an overview of lower magnification. Border colors indicate respective ANOVA complexity groups. **(J)** Fraction of stomata targeted by fungal hyphae. Box plot shows the scatter of data (solid line: median; dotted line: mean; whiskers: 5th/95^th^ percentile). The number of evaluated stomata is shown, with the number of evaluated images in brackets. For conifers, images of one year old needles were used. **(K)** Relationship between the fraction of stomata targeted by hyphae and leaf surface texture complexity based on linear regression analysis performed in SigmaPlot v.11. Data are shown as averages with standard deviation.

For the coefficients of determination statistics of environmental variables fitted to non-metric multidimensional scaling (NMDS) (e.g., Table 1) we used the R package vegan. We divided the approach into all tree species or split only to broad-leaved or coniferous tree species. For the input we used bray curtis distance matrices based on ITS- and 16S rRNA gene-based ASV abundances and for the leaf we used the surface texture complexity values and 13 parameters of leaf nutrients. Firstly, we performed a PERMANOVA (function adonis2). The p-values showed the significance of the permutation test and the R2 values indicated the percentage of microbiome variation explained by the respective parameter. For visualization we used the NMDS (Non-metric multidimensional scaling) with the function envfit in R to see if there is a strong correlation of the parameter to the community structure.

**Table 1:** Coefficients of determination statistics of environmental variables fitted to non-metric multidimensional scaling (NMDS) ordination of lichenized fungal community composition based on relative sequence read abundance data and nutrient contents from previous work [36]. The analysis was performed using the vegan package in R. Bold letters indicate statistically significant influences of the respective feature.

Fungi	*All Tree (n = 12)*		*Broad leaf (n = 8)*		*Needle (n = 4)*	
	*R* ^2^	*P*	*R* ^2^	*P*	*R* ^2^	*P*
Leaf surface texture complexity	0.4265	*0.091*	0.0603	0.9583	0.2735	0.515
Leaf surface texture complexity (ROI)	0.4640	*0.066*	0.0499	0.9583	0.2862	0.473
TOC	***0.6974***	**0.009**	**0.9847**	0.2083	0.1435	0.705
Nmin	0.2852	0.193	**0.6247**	0.6250	0.1130	0.677
Norg	***0.6167***	**0.009**	**0.6768**	0.5417	0.2028	0.496
C to N ratio	0.0681	0.743	**0.9864**	0.2917	**0.5331**	0.108
C to P ratio	0.3161	0.187	**0.6076**	0.6667	**0.5781**	0.127
N to P ratio	***0.6471***	**0.009**	**0.7947**	0.6667	***0.6538***	**0.048**
Ca	***0.6724***	**0.006**	**0.8078**	0.5000	0.4999	0.125
Fe	***0.5178***	**0.035**	**0.9960**	0.0833	0.2030	0.523
K	0.3925	0.117	0.2479	0.8750	**0.7009**	0.111
Mg	0.3261	0.185	**0.8325**	0.3750	0.2187	0.444
P	0.4665	0.060	0.4783	0.7500	***0.7477***	**0.031**
Bacteria	*All Tree (n = 12)*		*Broad leaf (n = 8)*		*Needle (n = 4)*	
	*R* ^2^	*P*	*R* ^2^	*P*	*R* ^2^	*P*
Leaf surface texture complexity	0.2904	0.251	0.3570	0.8750	**0.5904**	0.156
Leaf surface texture complexity (ROI)	0.3406	0.196	0.4023	0.8333	**0.7472**	*0.052*
TOC	***0.8545***	***0.001***	**0.9979**	0.0417	***0.7503***	***0.045***
Nmin	0.4189	0.114	**0.8288**	0.4583	0.2901	0.515
Norg	***0.7803***	***0.002***	**0.7493**	0.5833	0.3289	0.451
C to N ratio	0.2719	0.286	**0.9837**	0.0417	0.2510	0.571
C to P ratio	0.2937	0.230	0.4950	0.7500	0.2004	0.672
N to P ratio	***0.7821***	***0.005***	**0.8045**	0.3333	0.3050	0.517
Ca	***0.6872***	***0.007***	**0.9815**	0.1667	0.0541	0.903
Fe	0.4900	*0.075*	**0.8214**	0.5000	0.1994	0.659
K	0.0816	0.699	0.2724	0.8333	0.2810	0.478
Mg	***0.5984***	*0.036*	**0.6925**	0.6667	***0.7811***	***0.034***
P	0.4609	0.094	0.3908	0.7917	0.0983	0.821

### Structural equation modeling

The relationships between the leaf textural structure complexity and the bacterial and fungal community composition were analyzed using a structural equation model. The model evaluated used the variables: median complexity, the average size of stomata, the leaf type (broad-leaved vs. conifer), the Ellenberg T indicators, and the Shannon diversity indexes computed for bacteria and for fungi separately. In the model, leaf surface texture complexity was represented by a latent variable, related to the metrics median complexity and the Shannon diversity index of bacteria and fungi. The structural equation modelling was based on the sem 3.1–16 package in R.

### Visualization

For the boxplots we used the definition of the whiskers (Q1-1.5*IQR; Q3-1.5*IQR, Q1 = 0.25 Quantile, Q3 = 0.75 Quantile, IQR = Inter Quartile Range). Graphics were drawn using R or SigmaPlot v. 11.

## Results

We followed a hypothesis-driven correlative approach, firstly developing an abstract score quantifying abaxial leaf surface texture complexity of 21 broad-leaved tree species and six needle-leaved conifers (S1 Table). Secondly, the leaf surface texture complexity was tested for its dependence on leaf morphological features and habitat preferences (S2 Table). Thirdly, the contribution of leaf surface texture complexity to interactions with other organisms was investigated. We hypothesize that genetic determinants of leaf morphology influence the leaf surface structural complexity and that this complexity, in turn, affects interactions with other organisms.

### A quantitative surface texture complexity score allows cross-species comparisons

The surface texture complexity was derived as a comparative value from grayscale texture patterns on scanning electron microscopy images of the leaf abaxial side (S1 Fig). The apparent differences in structural patterns were systematically extracted as abstract features and resulted in quantitative relative ranking of the 27 tree species from low texture complexity (i.e., *Ulmus glabra*, *Fagus sylvatica* and *Larix decidua*) to high texture complexity (i.e., *Sorbus aucuparia* and *Prunus avium*) (Fig 1B, C, D). The surface texture complexity value is an abstract value based on features extracted from the microscopic images (S2 Fig), such as the combination of information density (measured via compression capabilities), textural heterogeneity (inverse of GLCM angular second moment), and local structural variability (gradient standard deviation). Complex textures thus exhibit low compressibility, non-uniform spatial patterns, and high variation in local intensity gradients. Despite this abstract value, leaves of tree species with high surface texture complexity (e.g., from *Prunus* or *Pyrus*) were visibly characterized by multilayered structures and wrinkles, wax papillae, and wax plates. By contrast, leaves of lower surface texture complexity (e.g., from *Ulmus*) showed more uniform cuticular wax patterns.

The resulting ranking of leaf surface texture complexity showed high accuracy (96%) in separating tree species. Variance in surface texture complexity (low variance: *Sorbus aucuparia* and *Prunus avium, Pyrus achras*; high variance *Ulmus glabra, Betula pendula*, and *Fraxinus excelsior*) was mainly due to stochastic occurrence of leaf surface features, such as the spatial organization of stomata, the stomatal di-morphism (e.g., *Prunus avium*), and other structural components like trichomes or glands. To our surprise, 60% of the broad-leaved species have dimorphic stomata (i.e., stomata of different size), most pronounced in *Prunus avium*. Dimorphic stomata were located on veins and originated from earlier development in the process of leaf expansion, where veins are formed prior to the mesophyll [46]. The calculated surface texture complexity values were robust against independent ranking runs, and also when limiting the calculation to the stomata as area of interest near stomata (S3A Fig). To assess batch-to-batch variations (e.g., from leaves collected throughout the season), for three species (*Acer pseuoplatanus*, *Fagus sylvatica*, *Quercus robur*) we calculated separate complexity ranks for each of the respective batches (S3A Fig). The complexity ranking of individual batches was within total variation for the respective species. Thus, in the following, we used the complexity ranking for each species involving all images from the whole leaf area averaged across all leaf samples. A feature leave-one-out analysis confirms that no single feature dominated the ranking (Spearman ρ = 0.86 to 0.97), with image compression size contributing most to discriminative power (S3B Fig, S2 Table). The leaf surface texture complexity ranking shows high robustness. Bootstrap resampling (n = 20, 80% samples) yields near-perfect stability (Spearman ρ = 0.998 ± 0.001), with 19 of 27 species showing zero rank variance (S3C Fig).

### Abaxial leaf surface texture complexity may contribute to leaf surface microenvironment

Tree taxonomy did not strictly relate to the observed leaf surface texture complexity (S4 Fig), i.e., closely related species can display highly different surface structure patterns (e.g., *Acer* species). In broad-leaved species, leaf surface texture complexity showed a positive correlation with leaf angle (r^2^ = 0.42, p = 0.02; multivariate regression analysis): leaves with a more horizontal angle showed lower leaf surface texture complexity. By contrast, in conifers, stomatal density (r^2^ = 0.85, p = 0.02), but not stomatal pore size, showed a positive correlation with leaf surface texture complexity (S5A Fig). Interestingly, Ellenberg temperature habitat indicator [35], but not light indicator or moisture indicator showed a considerable correlation with leaf texture complexity in broad-leaved species as well as in conifers (r^2^ = 0.36, p = 0.08 for broad-leaved species; r^2^ = 0.95, p = 0.16 for conifers; multivariate regression analysis; S5B Fig). This suggests that epidermal surface features, in addition to tree architecture and leaf anatomy, may be affected by the microenvironment of the leaf and thus may be an evolutionary parameter indicating the preferred growth habitat of the species origin. Leaf temperature in turn has been shown to be a major factor for pathogens [47].

### Abaxial leaf surface texture complexity protects against pathogens and lichen formation

Many fungi, algae, and cyanobacteria can colonize leaves and needles as pathogens, saprophytes, symbionts or in the form of lichens. We used an existing data set of leaf microbial communities [6] on a subset of twelve tree species (8 broad leaf, 4 conifers) across the range of leaf surface texture complexity scores to test for interdependence of microbial communities with leaf surface properties (S3 Table) by a multivariate regression approach. The total number of 2450 ASVs in the used data set [6] contained a fraction of 2.2% as known fungal endophytes. Thus, the majority of leaf-associated fungi were epiphytic. A distinction is not possible for bacteria at this stage. Total richness of fungi was not correlated with leaf surface texture complexity (S6A Fig). However, we observed a negative correlation (r^2^ = 0.88, p = 0.02; multivariate regression analysis) of the total number of colonizing bacteria with leaf surface texture complexity of broad-leaved species (S6B Fig). Across all tree species, and for fungi as well as bacteria, higher leaf texture complexity significantly (p < 0.01; multivariate regression analysis) resulted in lower richness in specialists (Fig 2A), but not in generalists (S6C Fig). This relationship was largely driven by the significantly higher richness of specialists on coniferous species, especially *Picea*.

When separating fungal species by their functional ecotypes, we observed a considerable negative trend with leaf surface texture complexity only for the proportion of pathogens in broad-leaved species (Fig 2B), but not for other ecotypes (see S3 Table). Thus, our data suggests that the leaves having higher surface texture complexity had a tendency for fewer pathogens (r^2^ = 0.46, p = 0.14; multivariate regression analysis). For conifers, we observed a negative trend of the proportion of lichen colonization (r^2^ = 0.79, p = 0.14; multivariate regression analysis) with leaf surface texture complexity (Fig 2B). We propose that complex leaf epidermal surface structures could present an important means of protection against pathogens (broad-leaved) or heavy lichenization (conifers). The interaction of leaf surfaces with leaf-colonizing fungi and lichens was therefore investigated in more detail also with respect to nutrient sources for the microbiome.

### Leaf surface is nutrient-poor and requires organismic interactions for epiphyllic growth

Abaxial leaves and needles were found rich in covalently bound carbon (C) and oxygen (O) molecules but were poor in nitrogen (N) (S7 Fig). The element distribution was linked mainly to structural components: the stomatal pores themselves were more O-rich compared to guard cell surfaces (e.g., *Quercus*, S7A Fig). The atomic composition in needles from *Abies alba* shifted over time, reflecting a temporal succession (2, 4, and 8 years of needles on the tree) of co-occurring fungal hyphae, which were visible by their more O-rich surface chemistry compared to the needle surface wax layer (S7B to D Fig). Indeed, fungal hyphae showed higher N content compared to the needle background, reflecting chitinous cell walls of fungi and/or glycan molecules in N-fixing cyanobacteria. Leaf-associated fungi were frequently found in direct physical neighborhood with algae and cyanobacteria (Fig 3). Such observed partnerships can reflect different organismic interaction types, ranging from parasitic interactions by phagocytosis (Fig 3A), mutualistic by aggregation (Fig 3B,C,D), or symbiotic by encapsulation of cyanobacteria or algae (Fig 3E,F,G), representing initial stages of lichenization. Initiating at the epistomatal wax layers of the stomatal antechamber, the fungal structures over time formed dense networks (Fig 3H), which in the form of lichens ultimately covered whole branches (Fig 3I).

We examined the influence of leaf surface texture complexity and nutrient content on fungal and bacterial community composition across 12 tree species using regression models (Table 1). Leachable total organic C, organic N, the N-to-P ratio, and Ca concentration in host leaves were significantly correlated with both fungal and bacterial community composition, indicating their influence on the colonizing microbiome. In coniferous trees, P concentration was significantly correlated with the fungal community, while Mg concentration was significantly correlated with the bacterial community (Table 1). Interestingly, in conifers, leaf surface texture complexity values calculated around regions of interest (ROI) at stomata showed an R^2^ value 0.74 (p = 0.052; multivariate regression analysis) in the correlation with bacterial communities, suggesting the contribution of surface texture complexity to bacterial community composition was in a similar magnitude as total organic carbon (TOC) or magnesium. For fungal community composition, the highest contribution of leaf surface texture complexity was found across all tree species (R2 = 0.42/0.46; p-value = 0.091/0.066; multivariate regression analysis). We conclude that while the nutrient composition of the leaf turned out as important factor defining the leaf microbiome, the surface texture complexity turned out as additional component not to be neglected, especially in conifers.

### Stomata as targets of fungal growth and initiation of lichen formation

Most fungal colonizers organized their proliferation towards stomata, irrespective of whether tree species were broad-leaved or coniferous (Fig 4A–I). Moreover, a considerably higher frequency of stomatal invasions was observed on leaves with a lower leaf surface texture complexity score (Fig 4J, K). We therefore conclude that leaf surface texture complexity plays an important role in protecting stomata structures from access by fungal colonizers. This relationship was found to be more pronounced in the needles of coniferous trees (Fig 4K), in which microbial colonization clearly initiated at the stomatal antechambers. Apparently, the leaf surface contains structures, for which functions in regulation of leaf microenvironment (S4 Fig) or in protection against (potential pathogenic) invasion of stomata (Fig 4) can be suggested.

## Discussion

Our work provides evidence that microscopic structures on the lower leaf surface significantly affect organismic interactions with leaf-colonizing microbes, particularly in defense against plant pathogens. These relationships became evident through the here developed surface texture complexity scoring based on microscope images. The surface texture complexity scores then allowed quantitation and ranking of leaf epidermal surface traits and assessment of their functional role in context of microbial colonization.

### Determination and ranking of leaf surface texture complexity

Texture, as defined as a statistical distribution of gray tones, has become increasingly used in plant sciences for the classification of image features. Since texture depends on spatial scale, smooth textures at larger scales become rough as the scale decreases, e.g., in microscope images [22]. This can be overcome by computing gray-level co-occurrence matrices (GLCM) from which typical texture features such as energy, entropy, contrast, absolute value, inverse difference, and homogeneity can be extracted and used as feature vectors for machine learning models. Based on isotropic GLCM, a 90% accuracy can be achieved for plant species classification [19, 20]. For denoising and decreasing the inter-class variation, typically wavelet and fractal dimensions are used during the extraction of texture features [48]. This successfully increased the prediction accuracy of plant species based on leaf surface patterns [20].

Based on previously published methods of feature extraction from images, our workflow was built on four steps: sub-image sampling, feature computation, species classification, and complexity ranking (details see Methods). The ranking based on the game theory approach allowed the quantitative ranking of different tree species by their leaf surface texture complexity values in an all-against-all comparison. We chose a game-theoretical framework due to mathematical flexibility across diverse datasets, its computational efficiency when scaling to larger species pools, and the intuitive clarity it provides when translating abstract texture data into a definitive hierarchical ranking, such the leaf surface texture complexity score developed here. Additionally, by treating each extracted segment as an individual during the ranking of the complexity traits, the game theory approach added to high intra-class robustness. The resulting quantitative ranking of leaf surface texture complexity values was used as a basis for analysis of functional hypotheses regarding whole plant abiotic and biotic parameters. In some species (*Betula*, *Fagus*) we observed a larger within-species variation of leaf surface texture complexity compared to other species (*Juglans*, *Prunus*). This may partly be attributed to different numbers of images available for each species, but further research is needed to carefully assess within-tree variation, for example in sun or shade leaves.

### Leaf surface texture complexity and environmental factors

A previous hypothesis for micro-scale cuticular surface structure proposed that uneven structures avoid the formation of water films [1]. Liquid water covering the stomata could significantly reduce the gas exchange of leaves through the stomata. In consequence, the exchange of CO_2_ with the atmosphere would be impaired and affect growth and survival of trees. The avoidance of water films and the associated establishment of epiphyllous microbial films of organisms is crucial particularly for the upper adaxial surface of leaves in terms of light harvest. However, here we analyzed the lower, abaxial surface of leaves, which in contrast to the upper leaf surface contains the stomata and which get fully wet only rarely, depending on leaf orientation. Interestingly, we observed a positive correlation of leaf surface texture complexity with leaf angle but also with Ellenberg temperature habitat indicator (S5 Fig). The Ellenberg temperature habitat indicator (Ellenberg T) is a numerical rating, typically ranging from 1 (cold-adapted alpine/arctic species) to 9 (warmth-loving Mediterranean species), that reflects the thermal requirements and distribution of a plant species relative to the heat gradient across Central Europe based on vegetation surveys. We therefore propose that leaf surface texture complexity indeed contributes to leaf microenvironments regarding leaf and/or stomatal moisture and temperature. It may well be possible that, depending on leaf orientation and anatomy, the leaf surface texture features may help buffering the leaf microenvironment against the surrounding, but this has not been measured at this point. It emerges that leaf surface structure may not as much regulate transpiration directly but rather affect microbial community establishment and the efficiency of pathogen entry, possibly through a component of affecting leaf surface temperature properties [47] and surface water availability.

### Stomata as entry pores for fungi

We frequently observed stomata to be penetrated by hyphae and stomatal antechambers as initiation points for microbial colonization. At this stage, it remains unknown how fungal hyphae detects the location of stomata. The total amount of water vapor emitted by stomatal cells exceeds the flux of CO_2_ by about a factor of 100 [49], suggesting moisture as a major determinant for stomata directed growth of hyphae. In addition, a large range of volatile organic substances are emitted at lower concentrations. Previously described overlap between epiphytic and endophytic communities can reflect surface-first colonization for some taxa [50] and movement of microbes from leaf surface and interior spaces via natural openings such as stomata. Thus, leaf surface traits (including stomatal microtopography) could plausibly influence also parts of the endophytic microbial community.

The source of nutrient acquisition of foliicolous lichens from host leaves remains unclear [51–53]. Apart from the N produced by the cyanobiont through atmospheric N_2_-fixation, mineral N from host leaves or needles has been suggested as an alternative or additional source of nutrients for the lichens. Mineral N (N_Mn_) and dissolved organic C (DOC) are water-soluble nutrient sources immediately available for surface-associated microbes. These nutrients can originate from atmospheric deposition (e.g., nitrate, ammonium; [54]), by a molecular film of liquid water that also allows ion transport across the cuticle [55], or by exploiting plant apoplasmic space after deterioration of the cuticle (Fig 4). The C-assimilation is driven by photosynthesis in the photobiont of the phyllosphere, which is highly dependent on water, light, and bioavailable N [56]. The differences in water potential between lichen thallus and host substrate, as well as within the lichen symbiont, provide a suitable environment for nutrient transport [57]. It has previously been suggested that fungi use resources excreted by the epidermal cells [7, 17]. However, the outer cell wall of epidermal cells is sealed by waxes of variable chemistry [18], and epidermal cells do not contain chloroplasts. Alternatively, it was proposed that cell walls of the stomatal cells are covered by a molecular film of liquid water that also allows ion transport across the cuticle [55]. We scanned the elemental composition of the leaf surfaces regarding C, O and N (S6 Fig) and could not detect any evidence for major nutrient leaching suggesting that leaf-associated organisms are required to be either symbiotic with cyanobacteria and algae, saprophytic, or pathogenic. In *Picea,* nutrient supply from the soil has been observed to affect the structure and form of cuticular wax polymers, which could in turn lead to variations in leaf surface texture complexity [58]. This provides interesting perspectives for future research, exploring leaf surface texture complexity across habitats of different nutrient availability.

Interestingly, growth and differentiation of *Uromyces appendiculatus*, a stomata-penetrating fungus infecting conifers (*Pinus*), was affected by surface topography, specifically by physical ridges of at least 0.5 µm. These minute barriers prevented fungal hyphae from finding the stomatal pore [59] supporting our findings of lower pathogenic growth on leaves of higher structural complexity (Fig 4). We can expect also other fungi to be able to distinguish unique patterns in the leaf surface during the colonization process, suggesting that surface features could have defensive functions. Moreover, increased fungal competition for nutrients as well as host defense mechanisms could lead to higher numbers of specialists, which was indeed observed for species with lower leaf surface texture complexity. Given that microbial community composition was available for only 12 out of the 27 the species for which leaf surface texture complexity was analyzed, these barely significant relationships between leaf surface texture complexity and leaf microbe community (Figs 2, Table 1) are remarkable and require further attention in future studies.

### Overall role of leaf surface texture complexity as assessed by structured modeling

After having presented multiple correlative relationships of environmental and organismic parameters with leaf surface texture complexity traits, a structured modeling approach quantified these relationships (S8A Fig). The structural equation model was fitted on the diversity and complexity data available for the limited species for which microbial data sets were available (S4 Table). The model confirmed the strong influence of the leaf type on the bacterial community composition, both directly and through the component of leaf surface texture complexity. The fraction of remaining unexplained variation was estimated at 46%. The leaf structural complexity thus had a considerable influence on the bacterial community composition but was not as strongly related to the overall fungal community composition. Comparatively, the Ellenberg habitat indicators had a much lower direct influence on the fungal community composition. The covariance between fungal and bacterial richness was very small, suggesting a weak linear relation between the diversity of these microbial kingdoms. By functional assignment of the fungal and bacterial community members, we identified the negative relationship between leaf surface texture complexity and pathogens or lichens (Fig 2B). It will be subject to future research to mechanistically verify these relationships. Clearly, this is a preliminary model approach that is limited by unknown variation in leaf surface texture complexity between trees. Variance of surface texture complexity values between species was found to be larger than leaf-to-leaf variation within one species (S2A Fig) suggesting non-random species-specific features. For *Picea*, the species with the highest number of identified leaf-associated microbes, the observed tree-to-tree variation in Shannon diversity indices was below 10% (relative standard deviation fungi 0.1; relative standard deviation bacteria 0.01; n = 5 trees). On average for other species, the tree-to-tree variation of the number of detected ASVs varied by 16% (S8B Fig).

Apparently, the leaf surface contains structures, for which our results point to functions in the regulation of leaf microenvironment or in protection against (pathogenic) colonization. According to the structural modeling, 54% of the variation in functional relationships could be explained by the factors considered here (S8A Fig, S4 Table). However, the leaf surface structural features may also not have a specific function at all. Such apparently non-functional structures exist, for example the structures of the leaf margin or the leaf form. Nevertheless, with respect to ecosystem biodiversity, the leaf surface and its structural texture complexity turned out to be an important trait potentially forming defense structures against numerous leaf-associated organisms. Interestingly, conifers were found with a remarkable high microbial richness and thus constitute a major component supplying space for organisms and ecosystem diversity.

Emerging evidence indicates that both leaf surface structural traits and leaf chemical composition influence the assembly and diversity of phyllosphere microbial communities, though they may operate through partly independent mechanisms. For example, variation in leaf surface microtopography such as vein and stomatal density has been shown to significantly correlate with bacterial community composition across tree species, suggesting that physical habitat complexity can regulate microbial colonization and stability on leaves [60]. In parallel, controlled experiments indicate that differences in leaf chemistry, driven by genetic variation and responses to herbivory, can alter phyllospheric fungal communities and overall microbial diversity, highlighting the role of chemical traits in host filtering and microbial selection [61]. These findings support a model in which structural and biochemical leaf traits jointly shape the phyllospheric microbiome, with structural complexity often influencing the physical niche space available to microbes and chemistry affecting nutritional and defensive environments, underscoring the multifactorial nature of host–microbe interactions in aerial plant surfaces.

It will remain an effort of future research to precisely identify the fungal species that are affected by structural barriers, and to separate fungal and bacterial functional groups in the modeling approach. Furthermore, regardless of being a huge analytical effort, in future it will be desirable to increase statistical power by increasing the number of observations, i.e., the number of species for which epiphyllic microbiomes were studied.

## Conclusions

We took a machine-learning based approach to quantify and rank epidermal surface texture complexity traits of European tree species. This approach of texture complexity quantitation can be generalized to other biological systems and processes. We propose that surface texture complexity should be taken in to account as a considerable species-based trait in defending against stomatal invasion and general microbial colonization, especially for pathogens. The occupation of the lower leaf surface may be detrimental to the leaf even though the organisms are not parasitic by growing into and blocking stomatal chambers. Although our study is observational in nature, it offers a new framework for understanding the role of surface morphology in plant-microbe interactions of tree species. Future experimental work will be essential to disentangle causal mechanisms and to study the variance of tree individuals. Nevertheless, this approach opens new avenues for exploring the ecological and evolutionary relevance of microscopic surface traits, with potential implications for biodiversity, ecosystem resilience, and tree health management in a changing climate.

## Supporting information

S1 Table List of species analyzed by SEM. (XLSX)

S2 Table Parameters of leaf surface features and texture complexity values. (XLSX)

S3 Table Species identified based on amplified sequence variants from [6]. (XLSX)

S4 Table Structural equation model fit statistics and parameter estimates for the fitted model. (XLSX)

S1 Fig SEM images of leaf abaxial surfaces. Representative images of the major species used in this study.
(PDF)

S2 Fig Determination of Leaf Surface Texture Complexity. (**A**) Workflow of quantification of leaf surface texture complexity as described in Materials and Methods. (**B**) Game theoretic approach for constructing the leaf surface texture complexity scores.
(PDF)

S3 Fig Variation of complexity ranking. **(A)** Complexity value of the different species as calculated independently based on image samples from the whole leaf (complexity 1, complexity 2) or focused on the stomata area (complexity ROI). Complexity ranking was performed separately for two different batches of leaves (yellow squares), which was available for *Acer pseudoplatanus*, *Fagus sylvatica*, and *Quercus robur*). Asterisks indicate species, for which molecular data of colonizing microorganisms were acquired. **(B)** Rank trajectories under feature leave-one-out analysis. High stability across configurations (ρ = 0.86–0.97) indicates no single feature dominates the ranking. **(C)** Bootstrap rank distributions (n = 20, 80% samples) with 70% of species show zero rank variance, with overall correlation ρ = 0.998 ± 0.001, indicating high robustness.
(PDF)

S4 Fig Mapping of leaf surface texture complexity groups onto a phylogeny of tree species. The phylogeny was reconstructed based on published phylogeny of vascular plants [62].
(PDF)

S5 Fig Relationship of anatomic and environmental factors with leaf surface complexity. (**A**) Correlations of leaf surface texture complexity with angle, and stomata morphology. (**B**) Relationship of Ellenberg indicator values of growth habitat characteristics (moisture indicator, light indicator, temperature indicator) with leaf surface texture complexity. Indicator values were obtained from published data sets [35]. Data points represent averages with standard deviation. P-values and r^2^-values were derived from a multivariate regression analysis using Excel Data Analysis Tool Pack.
(PDF)

S6 Fig Relationship of fungal and bacterial richness with leaf surface texture complexity. (**A**) Relationship of richness of epiphyllic *Basidiomycetae* and *Ascomycetae* with leaf surface texture complexity. (**B**) Relationship of richness of epiphyllic bacteria with leaf surface texture complexity (**C**) Correlation of fungal and bacterial generalists with leaf surface complexity. Data points represent averages with standard deviation. Species richness was assessed by amplicon sequence variants (ASV). P-values and r^2^-values were derived from a multivariate regression analysis using Excel Data Analysis Tool Pack.
(PDF)

S7 Fig Leaf surfaces are poor in nitrogen, as exemplified by surface element analysis. (**A**) *Quercus robur*. (**B**) *Abies alba,* 2 years. (**C**) *Abies alba*, 4 years. (***D***) *Abies alba*, 8 years. Scanning electron microscopy image (EVO15, Carl Zeiss Microscopy GmbH), and false-color images of elemental abundance (weight%) as determined Energy Dispersive X-ray Spectroscopy (EDS). Bar graphs show weight% of C, O, and N at areas with hyphae (black) or stomata (blue) and a reference region (white).
(PDF)

S8 Fig Structural equation model relating the leaf structural complexity (as a latent variable) to the diversity in bacteria and fungi and abiotic traits. (**A**) Structured equation model in which numbers represent model estimates, higher values representing more influential relations than lower values. Absolute values larger than 1 are highly significant (Supplementary Table S5). (**B**) Tree-to-tree variation of the number of identified amplicon sequence variants (ASVs) as average with standard deviation.
(PDF)
