# Supplementary material for: Complexity of leaf surface texture affects microbial colonization in temperate forest tree species
Source: PLoS One. 2026 May 29;21(5):e0349938. doi: 10.1371/journal.pone.0349938 (PMC13220997; doi:10.1371/journal.pone.0349938)

Supplementary Figure S1: SEM images of leaf abaxial surfaces. Representative images of the major species used in this study.

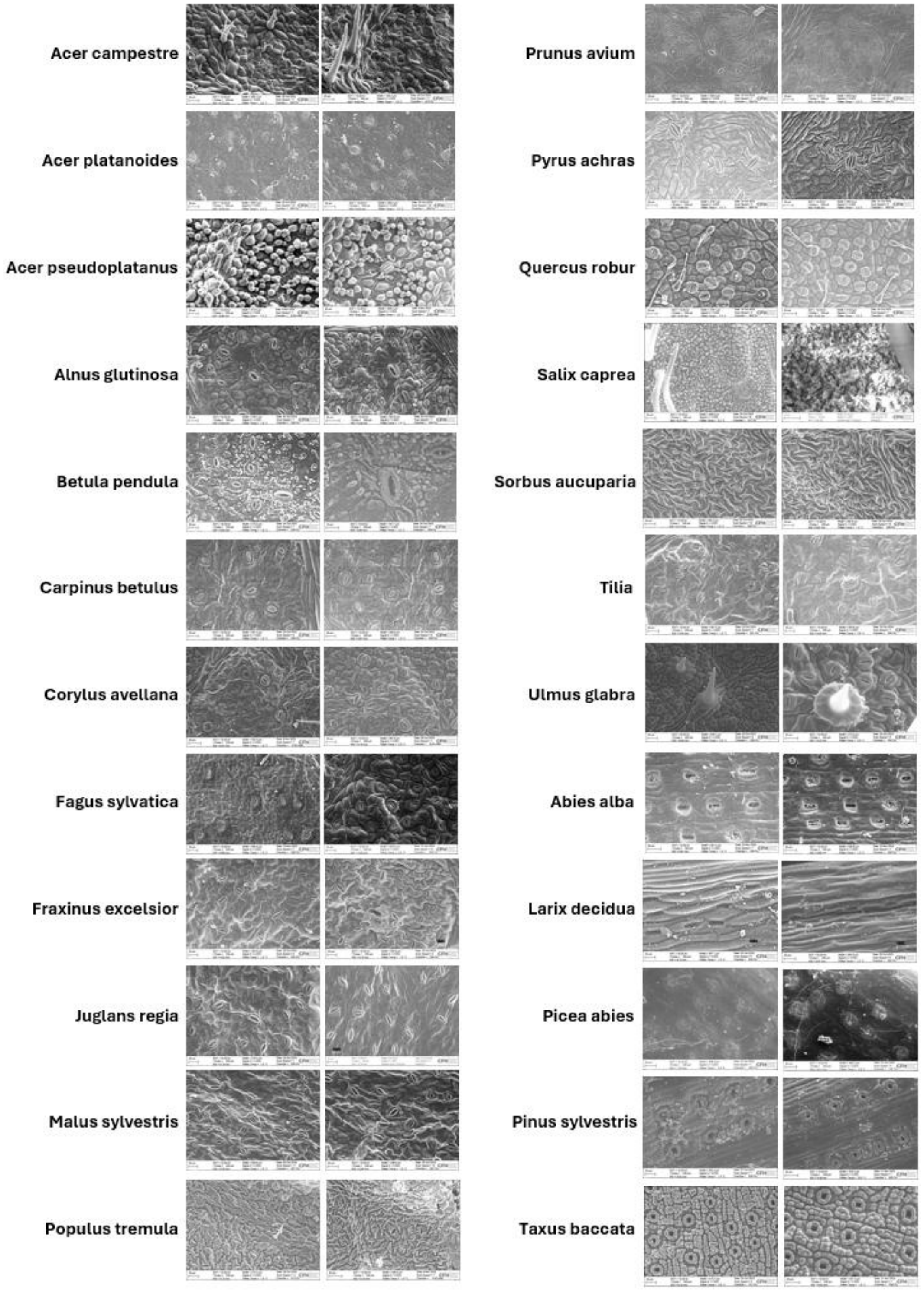

Supplement: S1 Fig — Representative images of the major species used in this study. (PDF) [file pone.0349938.s005.pdf]
