# Supplementary material for: Complexity of leaf surface texture affects microbial colonization in temperate forest tree species
Source: PLoS One. 2026 May 29;21(5):e0349938. doi: 10.1371/journal.pone.0349938 (PMC13220997; doi:10.1371/journal.pone.0349938)

Supplementary Figure S2: Determination of Leaf Surface Texture Complexity. (**A**) Workflow of quantification of leaf surface texture complexity as described in Materials and Methods. (**B**) Game theoretic approach for constructing the leaf surface texture complexity scores.

A

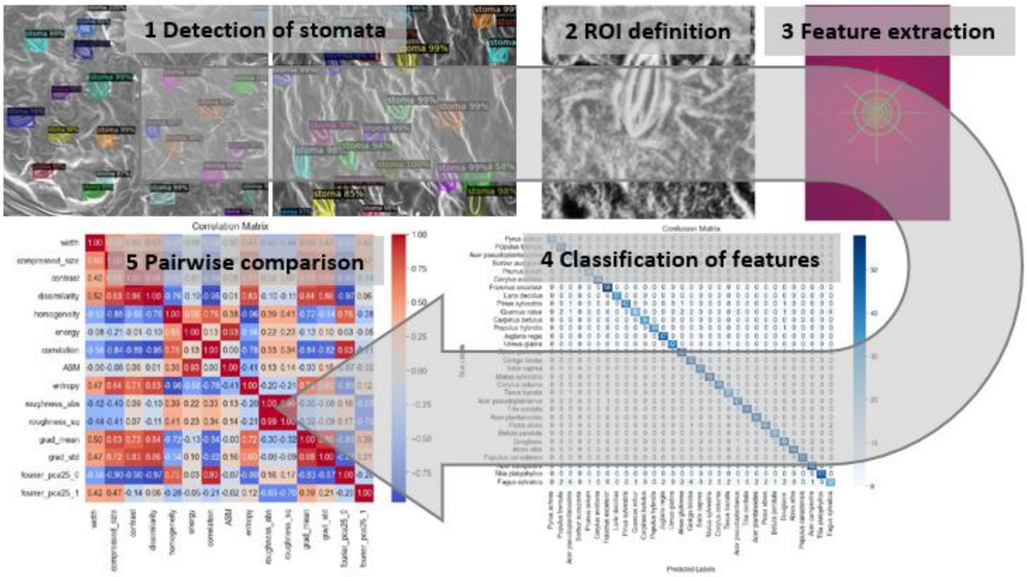

B

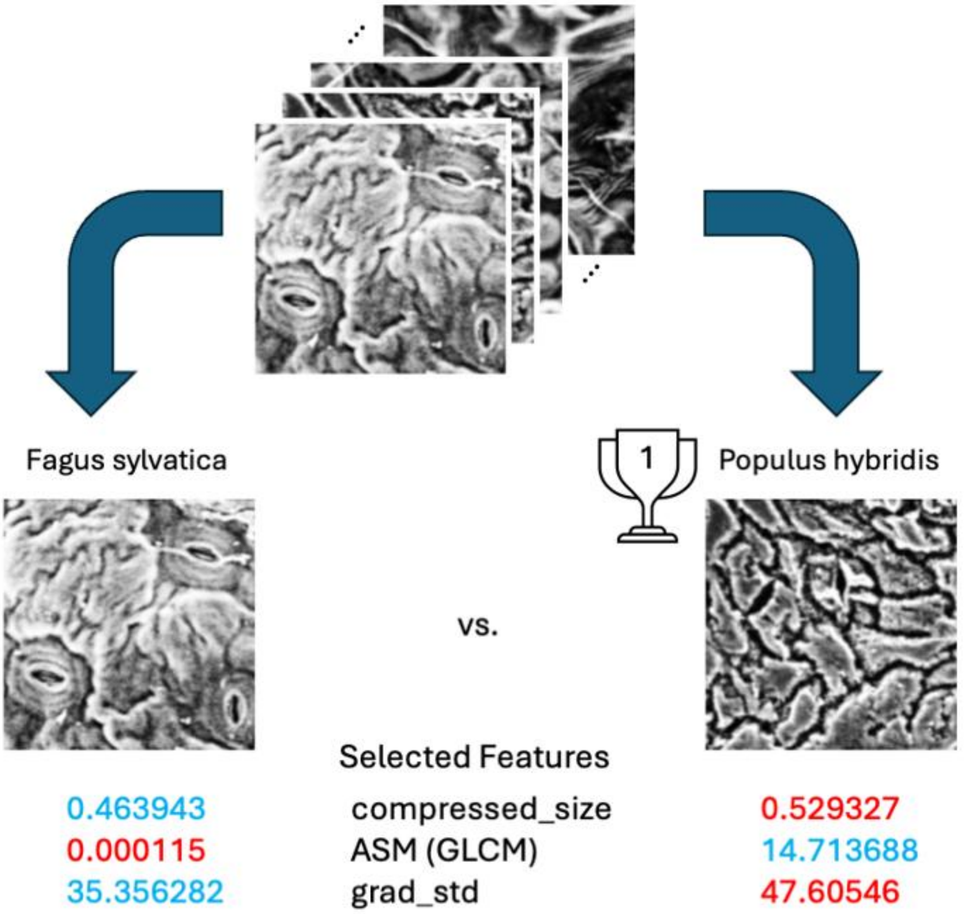

Supplement: S2 Fig — (A) Workflow of quantification of leaf surface texture complexity as described in Materials and Methods. (B) Game theoretic approach for constructing the leaf surface texture complexity scores. (PDF) [file pone.0349938.s006.pdf]
