# Supplementary material for: Complexity of leaf surface texture affects microbial colonization in temperate forest tree species
Source: PLoS One. 2026 May 29;21(5):e0349938. doi: 10.1371/journal.pone.0349938 (PMC13220997; doi:10.1371/journal.pone.0349938)

Supplementary Figure S3: Variation of complexity ranking. (A) Complexity value of the different species as calculated independently based on image samples from the whole leaf (complexity 1, complexity 2) or focused on the stomata area (complexity ROI). Complexity ranking was performed separately for two different batches of leaves (yellow squares), which was available for *Acer pseudoplatanus*, *Fagus sylvatica*, and *Quercus robur*). Asterisks indicate species, for which molecular data of colonizing microorganisms were acquired. **(B)** Rank trajectories under feature leave-one-out analysis. High stability across configurations (ρ = 0.86–0.97) indicates no single feature dominates the ranking. **(C)** Bootstrap rank distributions (n=20, 80% samples) with 70% of species show zero rank variance, with overall correlation ρ = 0.998 ± 0.001, indicating high robustness.

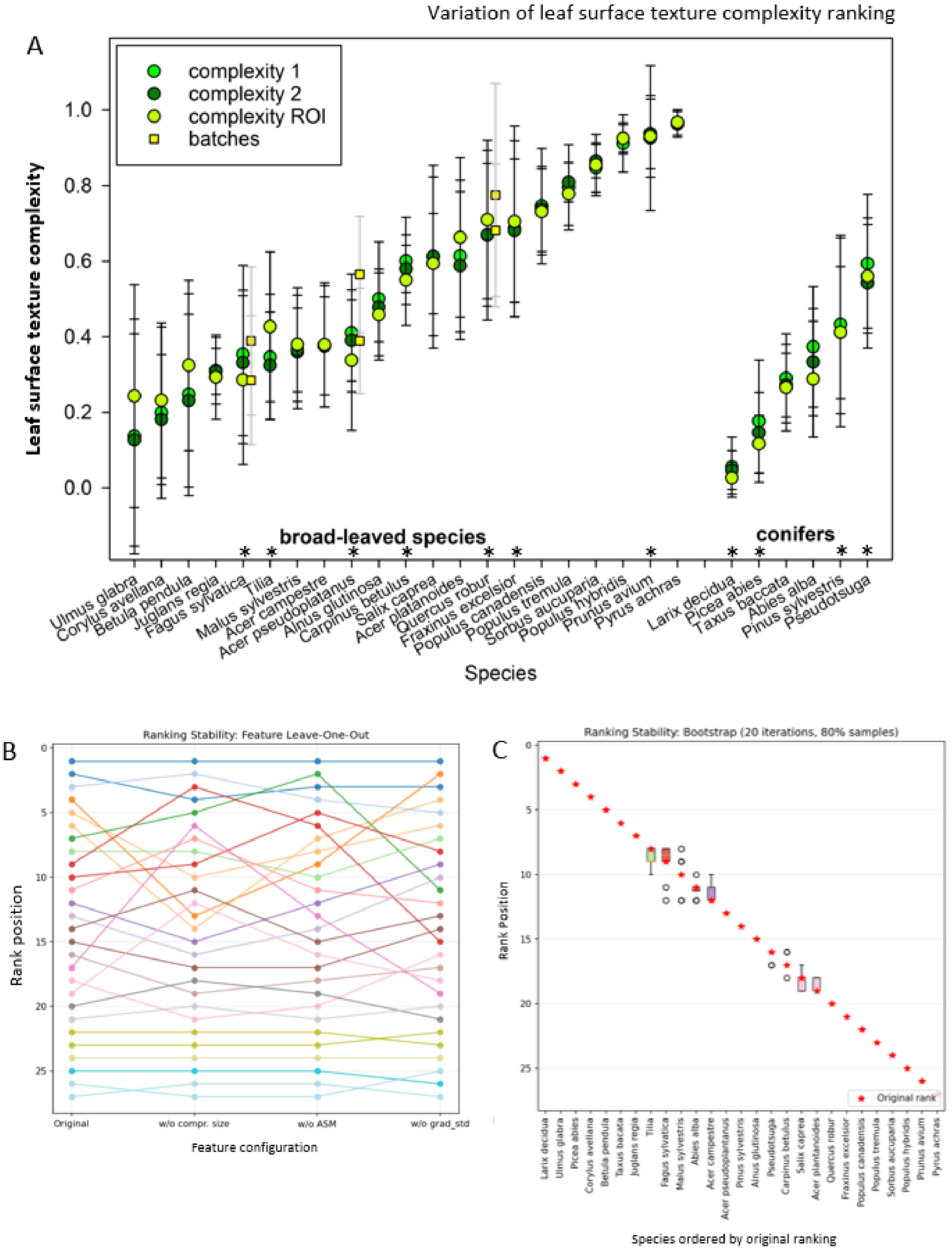

Supplement: S3 Fig — (A) Complexity value of the different species as calculated independently based on image samples from the whole leaf (complexity 1, complexity 2) or focused on the stomata area (complexity ROI). Complexity ranking was performed separately for two different batches of leaves (yellow squares), which was available for Acer pseudoplatanus, Fagus sylvatica, and Quercus robur). Asterisks indicate species, for which molecular data of colonizing microorganisms were acquired. (B) Rank trajectories under feature leave-one-out analysis. High stability across configurations (ρ = 0.86–0.97) indicates no single feature dominates the ranking. (C) Bootstrap rank distributions (n = 20, 80% samples) with 70% of species show zero rank variance, with overall correlation ρ = 0.998 ± 0.001, indicating high robustness. (PDF) [file pone.0349938.s007.pdf]
