# Supplementary material for: Complexity of leaf surface texture affects microbial colonization in temperate forest tree species
Source: PLoS One. 2026 May 29;21(5):e0349938. doi: 10.1371/journal.pone.0349938 (PMC13220997; doi:10.1371/journal.pone.0349938)

Supplementary Figure S4: **Mapping of leaf surface texture complexity groups onto a phylogeny of tree species.** The phylogeny was reconstructed based on published phylogeny of vascular plants (Durka and Michalski, 2012).

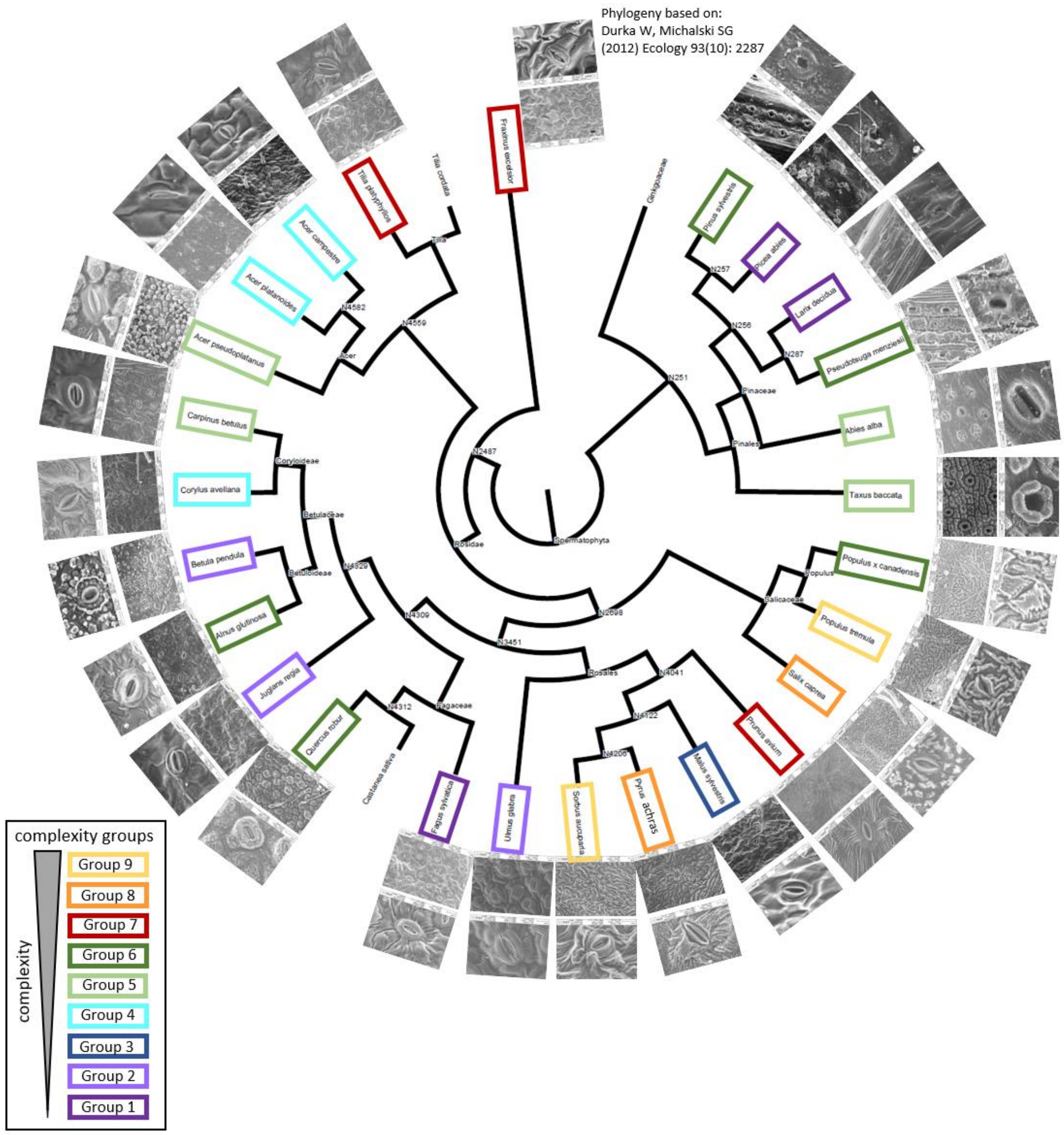

Supplement: S4 Fig — The phylogeny was reconstructed based on published phylogeny of vascular plants [62]. (PDF) [file pone.0349938.s008.pdf]
