# Supplementary material for: Complexity of leaf surface texture affects microbial colonization in temperate forest tree species
Source: PLoS One. 2026 May 29;21(5):e0349938. doi: 10.1371/journal.pone.0349938 (PMC13220997; doi:10.1371/journal.pone.0349938)

Supplementary Figure S5: **Relationship of anatomic and environmental factors with leaf surface complexity.** (**A**) Correlations of leaf surface texture complexity with angle, and stomata morphology. (**B**) Relationship of Ellenberg indicator values of growth habitat characteristics (moisture indicator**,** light indicator, temperature indicator) with leaf surface texture complexity. Indicator values were obtained from published data sets (Tichy *et al.*, 2022). Data points represent averages with standard deviation. P-values and r^2-values were derived from a multivariate regression analysis using Excel Data Analysis Tool Pack.

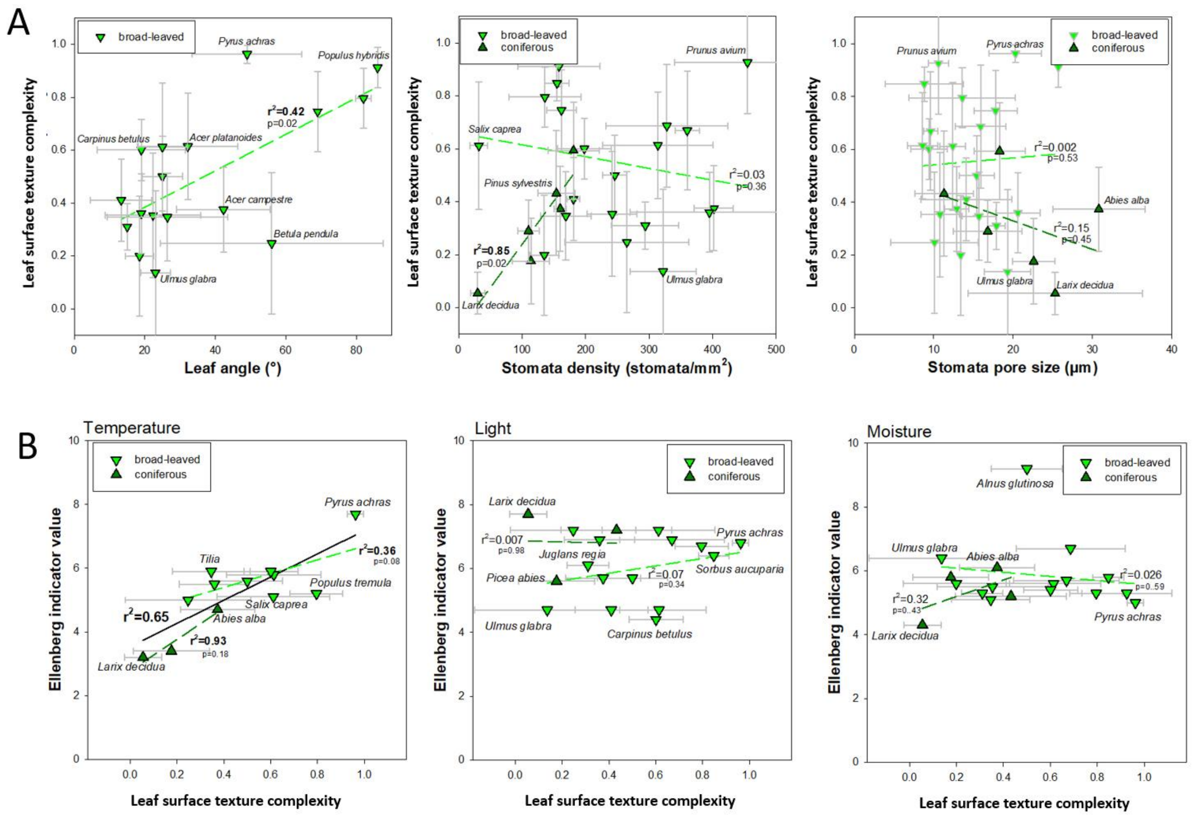

Supplement: S5 Fig — (A) Correlations of leaf surface texture complexity with angle, and stomata morphology. (B) Relationship of Ellenberg indicator values of growth habitat characteristics (moisture indicator, light indicator, temperature indicator) with leaf surface texture complexity. Indicator values were obtained from published data sets [35]. Data points represent averages with standard deviation. P-values and r2-values were derived from a multivariate regression analysis using Excel Data Analysis Tool Pack. (PDF) [file pone.0349938.s009.pdf]
