# Supplementary material for: Complexity of leaf surface texture affects microbial colonization in temperate forest tree species
Source: PLoS One. 2026 May 29;21(5):e0349938. doi: 10.1371/journal.pone.0349938 (PMC13220997; doi:10.1371/journal.pone.0349938)

Supplementary Figure S6: **Relationship of fungal and bacterial richness with leaf surface texture complexity.** (**A**) Relationship of richness of epiphyllic *Basidiomycetae* and *Ascomycetae* with leaf surface texture complexity. (**B**) Relationship of richness of epiphyllic bacteria with leaf surface texture complexity (**C**) Correlation of fungal and bacterial generalists with leaf surface complexity. In (A) and (B) data points represent averages with standard deviation, in (C) data points represent individual trees. In all cases, p-values were derived from multivariate regresson analysis. Species richness was assessed by amplicon sequence variants (ASV). P-values and r^2-values were derived from a multivariate regression analysis using Excel Data Analysis Tool Pack.

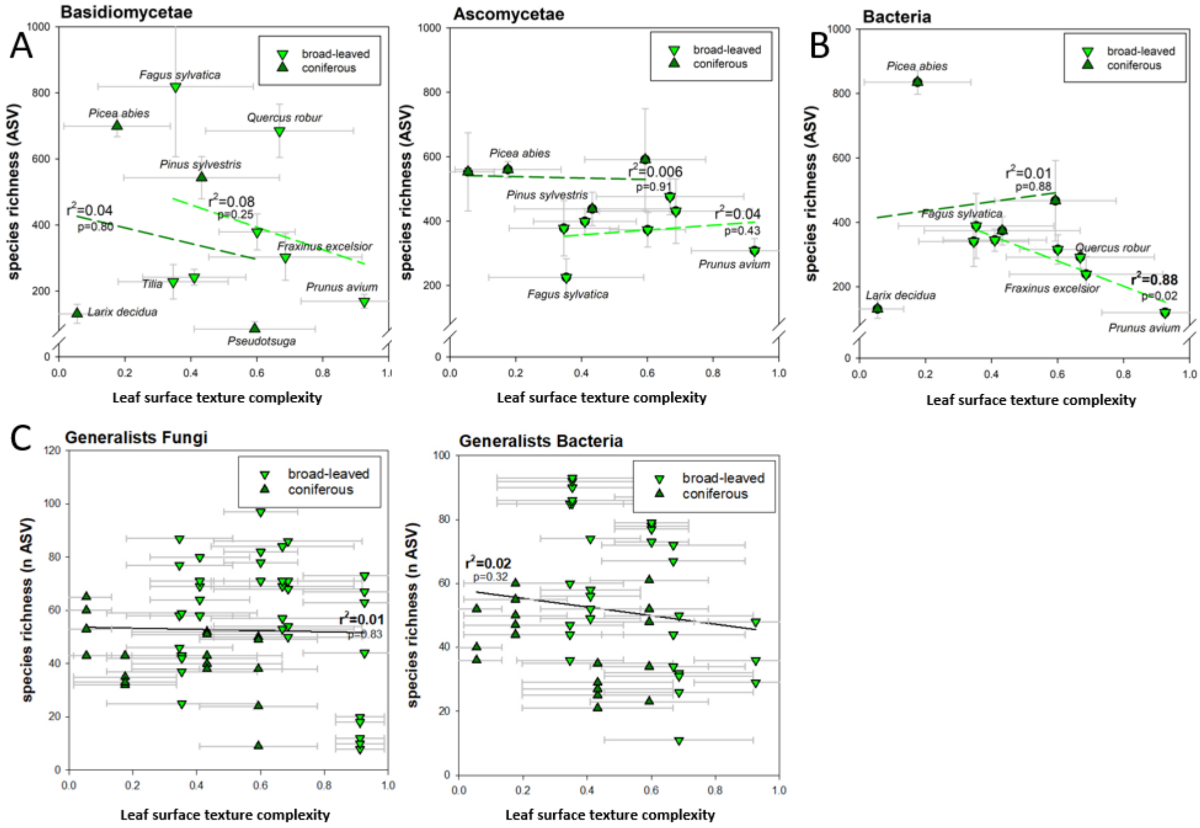

Supplement: S6 Fig — (A) Relationship of richness of epiphyllic Basidiomycetae and Ascomycetae with leaf surface texture complexity. (B) Relationship of richness of epiphyllic bacteria with leaf surface texture complexity (C) Correlation of fungal and bacterial generalists with leaf surface complexity. Data points represent averages with standard deviation. Species richness was assessed by amplicon sequence variants (ASV). P-values and r2-values were derived from a multivariate regression analysis using Excel Data Analysis Tool Pack. (PDF) [file pone.0349938.s010.pdf]
