# Supplementary material for: Complexity of leaf surface texture affects microbial colonization in temperate forest tree species
Source: PLoS One. 2026 May 29;21(5):e0349938. doi: 10.1371/journal.pone.0349938 (PMC13220997; doi:10.1371/journal.pone.0349938)

Supplementary Figure S7: **Leaf surfaces are poor in nitrogen, as exemplified by surface element analysis.** (**A**) *Quercus robur*. (**B**) *Abies alba,* 2 years. (**C**) *Abies alba*, 4 years. (***D***) *Abies alba*, 8 years. Scanning electron microscopy image (EVO15, Carl Zeiss Microscopy GmbH), and false-color images of elemental abundance (weight%) as determined Energy Dispersive X-ray Spectroscopy (EDS). Bar graphs show weight% of C, O, and N at areas with hyphae (black) or stomata (blue) and a reference region (white).

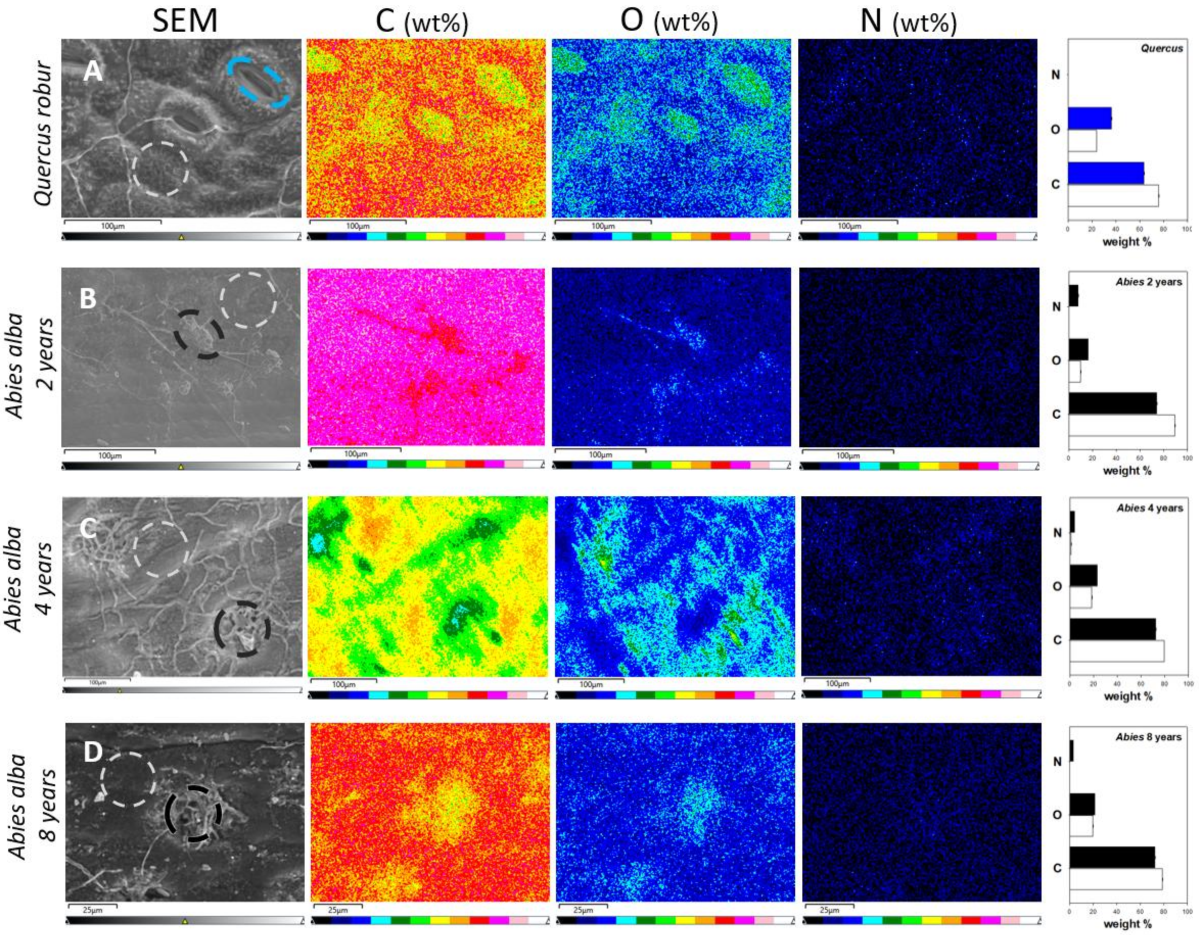

Supplement: S7 Fig — (A) Quercus robur. (B) Abies alba, 2 years. (C) Abies alba, 4 years. (D) Abies alba, 8 years. Scanning electron microscopy image (EVO15, Carl Zeiss Microscopy GmbH), and false-color images of elemental abundance (weight%) as determined Energy Dispersive X-ray Spectroscopy (EDS). Bar graphs show weight% of C, O, and N at areas with hyphae (black) or stomata (blue) and a reference region (white). (PDF) [file pone.0349938.s011.pdf]
