# Supplementary material for: Complexity of leaf surface texture affects microbial colonization in temperate forest tree species
Source: PLoS One. 2026 May 29;21(5):e0349938. doi: 10.1371/journal.pone.0349938 (PMC13220997; doi:10.1371/journal.pone.0349938)

Supplementary Figure S8: **Structural equation model** relating the leaf structural complexity (as a latent variable) to the diversity in bacteria and fungi and abiotic traits. (**A**) Structured equation model in which numbers represent model estimates, higher values representing more influential relations than lower values. Absolute values larger than 1 are highly significant (Supplementary Table S5). (**B**) Tree-to-tree variation of the number of identified ASVs as average with standard deviation.

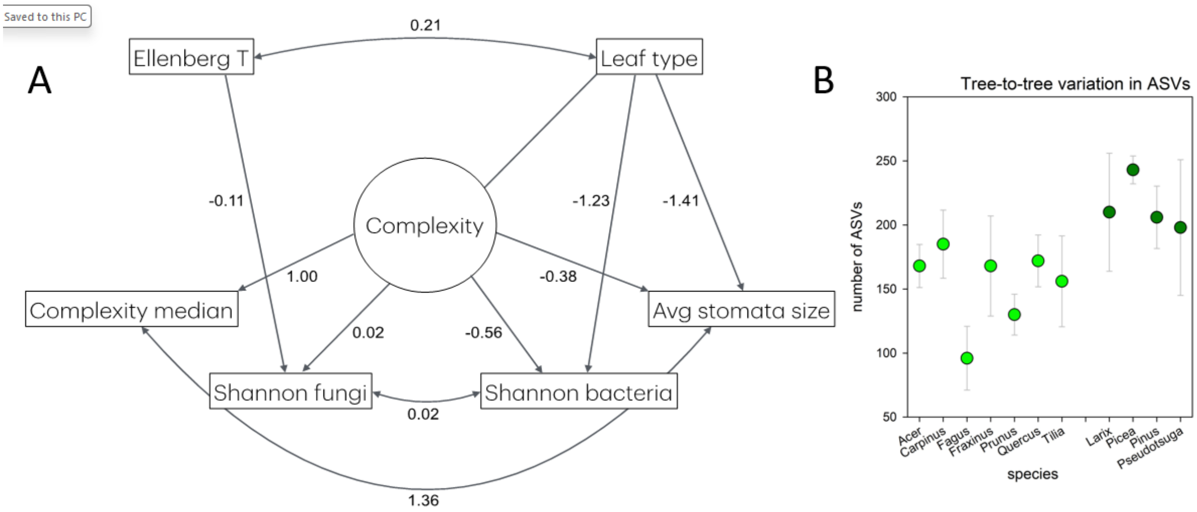

Supplement: S8 Fig — (A) Structured equation model in which numbers represent model estimates, higher values representing more influential relations than lower values. Absolute values larger than 1 are highly significant (Supplementary Table S5). (B) Tree-to-tree variation of the number of identified amplicon sequence variants (ASVs) as average with standard deviation. (PDF) [file pone.0349938.s012.pdf]
